# Ponder: Enabling Balloon-Borne Based Solar Unmanned Aerial Vehicle’s Take Off Diagnosis under Little Data

**DOI:** 10.3390/e24070997

**Published:** 2022-07-19

**Authors:** Yanfei Hu, Yingkui Jiao, Yujie Shang, Shuailou Li, Yanpeng Hu

**Affiliations:** 1 Institute of Information Engineering, Chinese Academy of Sciences, Beijing 100049, China; huyanfei@iie.ac.cn (Y.H.); lishuailou@iie.ac.cn (S.L.); 2 State Key Laboratory of Precision Measuring Technology and Instruments, Tianjin University, Tianjin 300072, China; jiaoyingkui@tju.edu.cn; 3 School of Physics and Electrical Engineering, Anyang Normal University, Anyang 455008, China; asyj0606@163.com; 4 School of Automation and Electrical Engineering, University of Science and Technology Beijing, Beijing 100083, China

**Keywords:** unmanned aerial vehicle, fault diagnosis, deep learning, generative adversarial network, little data

## Abstract

Balloon-borne based solar unmanned aerial vehicle (short for BS-UAV) has been researched prevalently due to the promising application area of near-space (i.e., 20–100 km above the ground) and the advantages of taking off. However, BS-UAV encounters serious fault in its taking off phase. The fault in taking off hinders the development of BS-UAV and causes great loss to human property. Thus, timely diagnosing the running state of BS-UAV in taking off phase is of great importance. Unfortunately, due to lack of fault data in the taking off phase, timely diagnosing the running state becomes a key challenge. In this paper, we propose Ponder to diagnose the running state of BS-UAV in the taking off phase. The key idea of Ponder is to take full advantage of existing data and complement fault data first and then diagnose current states. First, we compress existing data into a low-dimensional space. Then, we cluster the low-dimensional data into normal and outlier clusters. Third, we generate fault data with different aggression at different clusters. Finally, we diagnose fault state for each sampling at the taking off phase. With three datasets collected on real-world flying at different times, we show that Ponder outperforms existing diagnosing methods. In addition, we demonstrate Ponder’s effectiveness over time. We also show the comparable overhead.

## 1. Introduction

Near-space (i.e., 20–100 km above the ground) vehicles have been becoming the focus of research and developing in the world due to the strategic significance (e.g., dealing with various tasks such as high-resolution earth observation, communication relay, atmospheric research, and so on) in numerous fields and great flight condition (e.g., low atmospheric density and strong solar radiation) [1,2,3]. Low- speed near-space vehicles utilize conventional taxiing and taking off mode, thus it has to consume more fuel and take a longer time to climb to the desired height. This makes the research on near-space vehicles full of challenges, especially on power supply and structure design [4]. To overcome these challenges, rather than directly focusing on the power supply or structure design, balloon-borne based solar powered unmanned aerial vehicles have begun to be studied in the aviation field for its special taking off [3].

Different from traditional takeoff from runway and landing, BS-UAV’s take-off that is done with the help of a balloon’s rise up entails the cooperation of multiple phases and components. The concrete process of a balloon’s launch and take-off of BS-UAV is the following. The ball-borne based solar powered UAV is mounted by a high altitude balloon (the pitching angle of UAV is −90° at this moment), and when the balloon rises up to the altitude of near space, then aerostation releases the solar powered UAV. The UAV implements longitudinal pull-up control and enters the cruise flight stage, and is recovered by sliding and landing. The state of each component in each phase is of importance for the success of taking off for BS-UAV.

Even though the great advantages for taking off compared to traditional near-space UAV, BS-UAV encounters more fault due to (multiple components’ involvement in) its special takeoff mode and (light-level) structure design. The failure, especially before the pull-up phase, may lead to the crash of BS-UAV and a large cost on human property. Thus, timely diagnosing the overall state (e.g., normal or fault) by diving into the state of each component involved in the taking off of BS-UAV is of great importance. However, determining the overall state is challenging due to the characteristic of its taking off. First, the components involved in taking off process are diverse; as a result, there are numerous component faults or not an obvious fault for a component [5]. Second, the strong coupling relationship among each component poses a huge challenge for the overall diagnosing for BS-UAV’s taking off, and the multiple components further aggravate such coupling. This makes it time-expensive for technicians to analyze the fault source accurately real-time; especially, the failure on taking off may be caused by different components each time. Third, the data information for each component is large-scale due to continuous monitoring [6]. These all hinder the fast fault identification.

To overcome the above challenge, many techniques exist. At a high level, these techniques could be classified into three categories. The first is rule-based diagnosis. It diagnoses a fault depending on a rule base among each component; however, such kinds of methods become ineffective as the design or the architecture is more and more complex. As a result, a machine learning-based fault diagnosis method that learns the pattern hidden behind multiple data for diagnosing faults is proposed [7,8]. The learning process of a machine learning-based diagnosis model requires feature selection from expertise [3]. As a diagnosing model learns feature data’s distributions, different features that are utilized for a model lead to different diagnosing models. Thus, the human interference in feature selection is easy to cripple machine learning-based fault identification models. The third type is a deep learning-based diagnosis model [9,10,11,12]. Different from the human interference in feature selection, deep learning-based models [11] automatically learn a hidden representation on raw features based on predefined metrics and improve diagnosis accuracy. Deep learning-based models have presented multiple advantages in a fault identification domain.

Based on the above analysis, one feasible direction to accurately identify a fault in BS-UAV might be deep learning-based diagnosis models. The key idea of such kind of technique is to learn a model on the training data and use it to diagnose the fault in a real-time system once inputting a testing sample. The learning process of an effective diagnosis model requires plenty of data. However, actually, the data especially in a fault state are seldom due to the failure of taking off and thus the stop running or crash of each component. The lack of enough data in a fault state poses a critical challenge for applying such technique to timely determining the success of taking off or not.

In this paper, we propose Ponder, a deep learning-based fault diagnosis model combined with data complementation. More specially, Ponder complements fault data with a special generative network. This addresses the problem of a small amount of data in the fault state and allows us to diagnose the faults with a deep learning method in BS-UAV automatically. Deep learning learns a diagnosis model based on hidden representations that learns automatically from augmented training data, which allows us to diagnose fault real-time. We evaluated Ponder on the flying dataset, the evaluation results show that Ponder can identify the fault in BS-UAV’s taking off effectively. In addition, Ponder can effectively diagnose a fault in UAV as time goes by compared with the state-of-the-art diagnosing methods. Finally, we measure the overhead it introduces. To conclude, we make the following contributions:

(1) We introduce a novel data augmentation method to solve the problem of data unbalancing in fault diagnosing. This allows us to diagnose fault accurately in UAV with little fault data.

(2) We design a diagnosing model that combines data generating and a deep learning technique to diagnose faults in BS-UAV.

(3) We evaluate the effectiveness of Ponder on real-world flying experiment dataset. We show that Ponder significantly improves the performance of an existing fault diagnosing model.

The rest of the paper is organized as follows: Section 2 is the background. Section 3 analyzes our insight of data generation technique. Section 4 gives the overview and the details of our proposed Ponder. Section 5 is the experimental set and evaluation. Section 6 describes the discussion. Related works and conclusions are given in Section 7 and Section 8, respectively.

## 2. Background

In this section, we mainly discuss the background of deep learning based diagnosing method in mechanical field [13,14,15,16,17,18,19,20]. More details about BS-UAV (e.g., structure design or taking off manner) are shown in these works [1,2,3].

Research on fault diagnosis based on deep learning mainly includes Deep Belief Nets [9,21,22,23], Stacked Auto-Encoders [10], Recurrent Neural Networks [11], and Convolution Neural Networks [12]. DBN is a probabilistic generation model with a wide range of applications. It consists of multiple Restricted Boltzmann Machines layers, and there are connections between the layers but no connections within the layers. Training is divided into unsupervised pre-training and supervised back propagation fine-tuning for each layer. DBN is mainly used for feature extraction [24,25,26,27,28] and classification [29,30] in fault diagnosis. SAE is a multi-layer perceptual neural network composed of multiple self-encoding module units, which restores the original input to the maximum extent through the encoding and decoding process, and the model learns useful features by minimizing the differences between input and output, the model parameters are updated by the gradient descent algorithm, and the main functions of SAE are noise reduction filtering and feature extraction [3].

The processing units of RNN have both internal feedback and feed-forward connections, taking into account the correlation between samples, and can be used to process time series data or related data. RNN is often used in fault diagnosis for real-time fault diagnosis of complex industrial systems [9,11]. CNN is a feature extraction network composed of a convolutional layer and pooling layer alternately stacked, and then output by classification through the fully connection layer. It has the characteristics of sparse connection and weight sharing. Multiple convolution kernels perform convolution operations on the input to generate a convolution feature map. It is used for local feature extraction, combining abstraction layer by layer, and image recognition. CNN is very suitable for processing massive data [30,31], and fault diagnosis based on a CNN algorithm mainly uses CNN as feature extraction and recognition [32] or a classifier [25,29]. Most of these works focus on concrete design of a network structure but not the data challenges faced by them.

## 3. Insight of Ponder

Before demonstrating our method, we first introduce the insight of Ponder. As we know, the key aspect in Ponder is fault data generating. Thus, we focus on the insight of a generative adversary network. Indeed, the fault data are usually scattering around those normal data. We observe that the fault data for each component in the BS-UAV have activation bounds and cannot illustrate a meaningless value. Thus, we hold the view that the generated fault data should be dense around existing faults and sparse around normal data rather than arbitrarily scattering outside of the distribution of normal data. Based on this insight, we aim at generating fault data well aware. The key idea is to separate data of BS-UAV in the normal and fault state at first, and then generate more fault data around fault clusters and less around normal clusters. Considering that the data produced by each component are high-dimensional and such high-dimensional data make it difficult for clustering, we aim to compress high-dimensional data into a low-dimensional space.

## 4. Methodology of Ponder

We design Ponder to assist fault diagnosis in BS-UAV under the condition of unbalanced data. Ponder is short for “data comPlementatiON with Deep lEaRning based fault diagnosis model”. In the following, we first explain the overview of Ponder, followed by the technical details of each component.

### 4.1. Overview of Ponder

**Figure 1:**
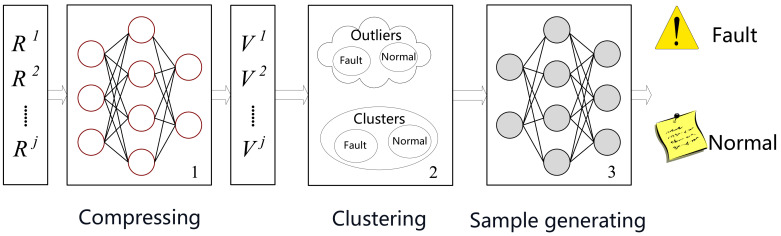
The overview of Ponder. Ponder consists of three modules. The first two are compressing network and clustering model, and the last is a model for fault data generating and fault diagnosing. $R^{j}$ and $V^{j}$ are the corresponding high dimensional raw data and low-dimensional representations.

We design the whole pipeline of Ponder, as shown in Figure 1. At a high level, Ponder mainly consists of three steps. The first step is pre-processing. In this step, we transform high-dimensional data into a compressed representation. The low-dimensional data allow us to cluster data in normal state and fault state accurately. In step2, we cluster on the compressed representation for separating different state’s data, which make it easy for generating fault data well aware. In step3, we generate fault data utilizing a generative network, which allows us to discriminate fault state accurately and timely.

**Pre-processing.** We transform high-dimensional data into low-dimensional representation. The low dimensional data allows us to perform accurate clustering. Given high dimensional data, we train an auto-encoder for compressing. Auto-encoder is a model with encoder and decoder. Encoder compresses high-dimensional data into low-dimensional representation, and decoder reconstructs high dimensional data based on the representation. More specifically, we train an auto-encoder with the goal that the high-dimensional data that decoder reconstructs is close or similar to original data. In this way, low-dimensional representation captures the corresponding high-dimensional features well and can be used for further clustering.

**Clustering.** We cluster data in normal states and that in fault conditions. The separation allows us to easily generate data around their distributions. Given the low-dimensional representations, we use a density-based clustering model called DBSCAN, and cluster existing representations into clusters. We use density-based clustering method with the following considerations. Density-based clustering method clusters data into normal state and fault state with the tolerance to abnormal data in dataset. These abnormal data form an outlier dataset and can be used as the reference for fault data generating. We do not utilize a K-means method since its clustering is sensitive to abnormal data. In addition, it is a distance-based clustering method and does not conform to our insights. In this step, we further cluster the outlier into a normal cluster and fault cluster.

**Data Generating.** We generate fault data depending on the clusters separated for further deep learning based fault identification. Given each cluster and the outlier, we train a modified generative adversarial network as our generating basis. Since we generate fault data based on clusters and outliers rather than learning existing distributions, this makes it different from the standard GAN. In this training, the generative adversarial network should be trained with the following rules. First, in areas near the cluster of fault data, we carefully expand the region of data and the expansion becomes less aggressive closer to data in normal conditions. Second, in the area of outliers, we obey the same rule based on an assumption that the outliers also obey each cluster’s distributions.

Therefore, the GAN we used has two generators due to the presence of outliers and clusters. In addition, a discriminator in the GAN network is needed to discriminate the generated data and that of original data. We will introduce the details of each component in the following.

### 4.2. Technical Details

In this section, we present the technical details of each component in Ponder. We start by key notations. Given an input dataset X,Y, where *X* denotes the set of normal samples and fault samples, *Y* corresponds to their labels. Within the dataset, each sample *x*∈$R^{1\times p}$ is a p-dimensional vector, and the sample’s label is represented by an integer value, indicating the corresponding state, i.e., *Y*∈{0,1}, where 0 indicates data in normal states and 1 denotes data in fault conditions.

#### 4.2.1. Auto-Encoder

Auto-encoder aims to transform high-dimensional data into low-dimensional representations to better identify the underlying clusterings. Based on the data in normal states, we want to learn an auto-encoder *f* to map any high-dimensional data *x* from *X* into *h* in a hidden space. As introduced in the overview part, the hidden space should maintain an accurate representation of raw data in high-dimension space. Thus, the loss for training our auto-encoder should be the following. Formally,

(1) $$\begin{array}{c} L_{auto}=\sum_{i=0}^{K}l_{f}\left(y_{i},\bar{y_{i}}\right),i\in \left[0,K\right] \end{array}$$

where $\bar{y_{i}}$ and $y_{i}$ are the high-dimension data output by auto-encoder and the original high-dimensional data in the dataset, respectively. We utilize MSE as our loss function $l_{f}$, and train our auto-encoder with the goal of minimizing the loss, $L_{auto}$.

Note that here we do not use fault data for the auto-encoder’s learning so that our auto-encoder may make the low-dimensional representation of fault data more separated. In the auto-encoder, the encoder’s network structure is a Multi-layer Perceptron (MLP) with multiple hidden layers, and the decoder is a reverse of the encoder. With auto-encoder, we transform the feature in original feature space into a compact representation *h* in a hidden space *H*.

#### 4.2.2. Dbscan

DBSCAN aims at clustering representations into normal and fault clusters for generating data based on them in the subsequent task. Concretely, first, it classifies representations into three categories with two parameters, $d_{r}$, $t_{no}$. $d_{r}$ is a distance semidiameter, and $t_{no}$ is the minimal number in the area of distance radius. The three categories of data point are core point, bound point, and outlier point. Then, DBSCAN classifies core point into different clusters. More concretely, it selects a core data point and assigns a cluster, and searches all the density connected points of this selected core point. It loops the selecting and assigning until the last point. Distance function is also a key point in determining the type of the data point, since DBSCAN needs to calculate $d_{r}$. We use $L_{2}$ norm as a distance function, i.e., Euclidean distance. We use a Silhoutte Coefficient as the clustering evaluation metric. Formally,

(2) $$\begin{array}{c} s(i)=b(i)-a(i)/max\{b(i),a(i)\},i\in [1,K] \end{array}$$

where a(i) is an average distance of sample *i* to the other samples in same cluster, and b(i) is an average distance of this sample to other samples in other clusters. Obviously, if the score of s(i) is close to 1, it illustrates that the sample *i* has been well classified, and 0 means it might be a bound point between each cluster, −1 denotes it is a sample of other clusters.

Finally, we cluster data *h* including normal state $h_{n}$ and fault state $h_{f}$ into clusters $h_{c}$($h_{nc}$ and $h_{fc}$) and outliers $h_{o}$($h_{no}$ and $h_{fo}$). Note that we choose the value of $t_{no}$ based on the rule of thumb that the value of $t_{no}$ is similar with the order of the dimension. For our case, we have data of 10 dimensions; thus, $t_{no}$ is 10.

#### 4.2.3. Modified Generative Adversarial Network

The network aims to generate fault data based on each cluster output by DBSCAN. Based on each cluster, we want to learn a modified generative adversarial network (MGAN) for completing fault data generating. To this end, we should determine the network structure first and then explore the loss for training such network.

**Figure 2:**
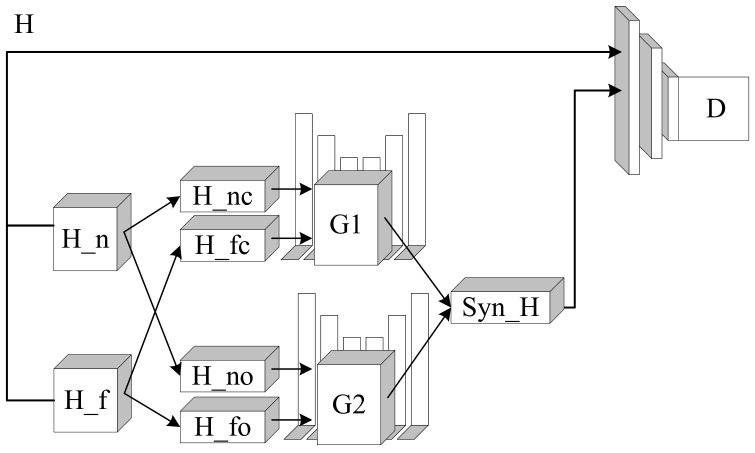
The details of MGAN. H_n and H_f are representations of normal state and fault state in hidden space. *c* and *o* denote clusters and outliers. *G* and *D* represent generator and discriminator. Syn_H means the synthetic data of fault state.

For determining the network’s structure, as illustrated in the overview, after clustering, the data have been classified into two clusters and two outliers. Thus, the fault data that need to be generated have two types. The first type is a distribution that conforms to the clustered distribution, and the second is a distribution that fits to the outlier area. Based on this analysis, the concrete structure of the modified generative adversarial network is shown in Figure 2.

Next, we explore the loss for the two generators. To make generative network learn the real distribution of fault data, when we learn the distribution in the clusters and outliers, we all expand the fault data region carefully around normal cluster or normal outlier regions. Specifically,

*Data Synthesis in the Cluster Region.* For learning the real distribution $d_{rc}$ conforming to (approximating) the data in clusters [33], as shown in the upper part of Figure 2, we train G1 to simulate this distribution, where G1 generates data in high-density regions. Concretely, if the probability of the data $\tilde{s}$ generated by G1 falling into the high-density regions of normal states is bigger than a threshold $p_{n}\left(\tilde{s}\right)>\lambda$, it will be generated with a lower probability; otherwise, it will generate data with a distribution balancing clustered data of normal states and fault states. We use α to control the generated sample to approximate normal or fault clusters. We define the distribution required as the following:

(3) $$d_{c}=\left\{\begin{array}{ccc} \; & \alpha p_{n}\left(\tilde{s}\right)+\left(1-\alpha \right)p_{f}\left(\tilde{s}\right) & ,p_{n}\left(\tilde{s}\right)\leq \lambda \wedge \tilde{s}\in H\\ \; & \frac{1}{\tau p_{n}\left(\tilde{s}\right)} & ,p_{n}\left(\tilde{s}\right)>\lambda \wedge \tilde{s}\in H \end{array}\right.$$

where τ is the normalization term, and λ is a threshold to indicate whether the generated data are in high-density normal regions. *H* denotes the whole hidden space.

In order to learn this distribution $d_{c}$, we minimize the KL divergence between defined distribution that we will learn and real distribution of clusters $d_{rc}$. The function is the following:

(4) $$L_{KL(d_{rc}||d_{c})}=-H\left(d_{rc}\right)+\underset{\tilde{s}∼d_{rc}}{E}\log P_{d_{c}}\left(\tilde{s}\right)$$

KL divergence is the expectation value of the logarithmic difference of $d_{rc}$ and $d_{c}$ on $d_{rc}$ distribution. After transformation, it can be divided into two parts. The first term is the opposite value of $d_{rc}$ entropy, and the second term is the expectation of $\log P_{d_{c}}\left(\tilde{s}\right)$ on the $d_{rc}$ distribution. $\log P_{d_{c}}\left(\tilde{s}\right)$ means the logarithmic of the probability of sample in $d_{c}$ distribution.

In addition, we should make the generated data in a fault condition and the real fault data indistinguishable. In other words, the generated data are located in a clustered region as far as possible. Thus, $L_{clus}$ is required as the following:

(5) $$L_{clus}=\left|\right|\underset{\tilde{s}∼d_{rc}}{E}f\left(\tilde{s}\right)-\underset{s∼h_{c}}{E}f\left(s\right){\left|\right|}^{2}$$

Here, *f* is the function of discriminator.

Overall, the loss for learning generator G1 is

(6) $$L_{G1}=L_{KL(d_{rc}||d_{c})}+L_{clus}$$

*Data Synthesis at Outlier Region.* As we have clustered the outliers into two clusters, the loss for second generator is the same as that of $L_{G1}$. *Discriminator.* Different from the commonly used generative adversarial network that aims at discriminating synthesized fault data from real fault data, the special designed discriminator aims to discriminate normal data from fault data. Concretely, it discriminates normal data from synthesized fault data, and from real fault data. The discriminator not only participates in the training process but also acts as the diagnoser in diagnosing steps. For learning this discriminator, we aim to optimize the following loss function:

(7) $$\begin{array}{cc} L_{disc}= & \underset{s∼p_{n}}{E}\left[\log D\left(s\right)\right]+\underset{\tilde{s}∼d_{rc}}{E}\left[\log\left(1-D\left(\tilde{s}\right)\right)\right]+\\ \; & \underset{\tilde{s}∼d_{ro}}{E}\left[\log\left(1-D\left(\tilde{s}\right)\right)\right]+\underset{s∼p_{n}}{E}\left[D\left(s\right)\log D\left(s\right)\right]+\\ \; & \underset{s∼p_{f}}{E}\left[\log\left(1-D\left(s\right)\right)\right] \end{array}$$

Here, the first three terms distinguish normal data from synthesized fault data. The fourth conditional entropy term is to recognize normal data with high confidence, which corresponds with our assumption. The last term encourages the discriminator to correctly classify normal data from real fault data. With all the terms, the discriminator could be learned to classify data in normal states from both real and synthesized fault data.

#### 4.2.4. Hyper-Parameters

The batch size is 128, and the training epoch is 250. We use the Adam optimizer for minimizing the loss of $L_{auto}$, $L_{G1}$ $L_{G2}$ and $L_{disc}$. The technical details of this optimization technique can refer to [34]. Here, the batch size and training epoch can be randomly selected. The larger the batch size, the better parameter learning. Note that we should control batch size value to avoid out of memory in training.

For auto-encoder, the encoder has two hidden layers. The input size has dimensions of 60, and the output size is 10 dimensions. We set the dimension size of two hidden layers as 30 and 20 dimensions, respectively. For MGAN, the discriminator and the generators are feed-forward neural networks. Each network of generators and discriminator contains hidden layers (with dimensions of 20 and 5, respectively). The dimension of noise for each generator is 40, and the output dimension of generators is the same as that of the auto-encoder, which is 10. The threshold λ is set as 0.95 of the probability of normal data predicted by a pre-trained probability estimator. We set τ to a small value 0.1. The last two settings of hyper-parameters (i.e., λ and τ) have already been shown their effectiveness in this work [33]. We use a pull-away term to minimize $H(d_{rc})$ [35] and the technique proposed by [36] that uses a neural network to approximate $p_{n}$. For the hyper-parameters, we use these default parameters and do not consider the sensitivity of them to model performance.

## 5. Evaluation

We evaluate the effectiveness of Ponder on fault diagnosis with the flying data of BS-UAV. The evaluation are performed on a server with two Intel Xeon E5-2630 v3 2.4 GHz CPUs (32 processors) and 128 GB memory. We focus on four key aspects: (a) design choice, (b) effectiveness, (c) robustness, and (d) overhead.

### 5.1. Experimental Setup

*Datasets.* We prepare three different datasets for evaluating Ponder in our experiment. Each dataset is made up of flying data of the same type of BS-UAV in normal conditions and fault condition, with 354,000 normal items and 6000 fault items (little fault data). Each sample is a matrix of 1 × 60. The time interval of three datasets is over half a year, which enables us to evaluate the robustness of Ponder over time. More specifically, they are collected at May 2019, February 2020, and October 2020. For convenience, we refer them as D-May, D-Feb, and D-Oct, respectively.

For each dataset, D-May is mainly used for training and testing. D-Feb and D-Oct are used for testing. More specifically, we randomly divide D-May into two parts, 70% of D-May is training dataset (Training), and the other is testing dataset (Testing). In addition, 20% of the samples in the training dataset are held out for validation.

*Baseline Models.* We utilize three existing methods as our baseline models. More specifically, for validating design choices, we assess the performance of Ponder with a deep learning based diagnosing model with one generator; their difference only exists in the number of generators. For evaluating Ponder’s performance, we compared Ponder with the state-of-the-art data generation techniques, ODDS [33]. ODDS works under the same situation as a few samples as ours. In addition, we also compare Ponder with machine learning based models that use Random Forest (RF) as the diagnosing algorithm. RF is a model with multiple groups of parameters or models and behaves best among machine learning based models. These baseline models allow us to evaluate Ponder thoroughly.

*Metrics.* As the essential of the fault diagnosing is a classification problem, we choose metrics widely used in a classification domain. These metrics include precision (the correct diagnosis among all the diagnosis determined by diagnosis model), recall (the true parts among the actual ones), and F1-score. F1-score indicates a comprehensive evaluation for the performance, which balances precision and recall: F1=2×Precision×Recall/(Precision+Recall). In addition, we also utilize false positive rate (FPR) that equals 1−recall as our evaluation metrics. We choose them (e.g., precision, recall, F1-score and FPR) as the final measurement for the performance of each diagnosing model if not otherwise stated.

*Training and Testing.* Generally, we use training dataset for learning each model’s parameters, and use validation dataset for deciding their best hyper-parameters. We test each model on a testing dataset and evaluate each model’s performance by calculating each metric based on a testing dataset.

Specifically, for building Ponder, first, we use all normal data in dataset Training and learn the parameters of auto-encoder, and perform dimension compressing on each sample in dataset Training by using a learned auto-encoder. Then, we cluster on all low-dimensional representations in dataset Training into four clusters. The four clusters include two clusters of normal data and fault data, and two clusters of outliers of normal data and fault data. Third, we train the generator based on normal clusters and outliers. For ODDS, the building procedure is the same except the lack of clustering for outlier samples. For RF, we directly train them on dataset Training.

For testing Ponder, in other words, diagnosing if there exists a fault using Ponder, we import each sample of testing dataset into the above learned generator. The outputs of generator are the diagnosed result. Fault diagnosis using ODDS and RF are the same steps except for importing each sample into their models.

**Figure 3:**
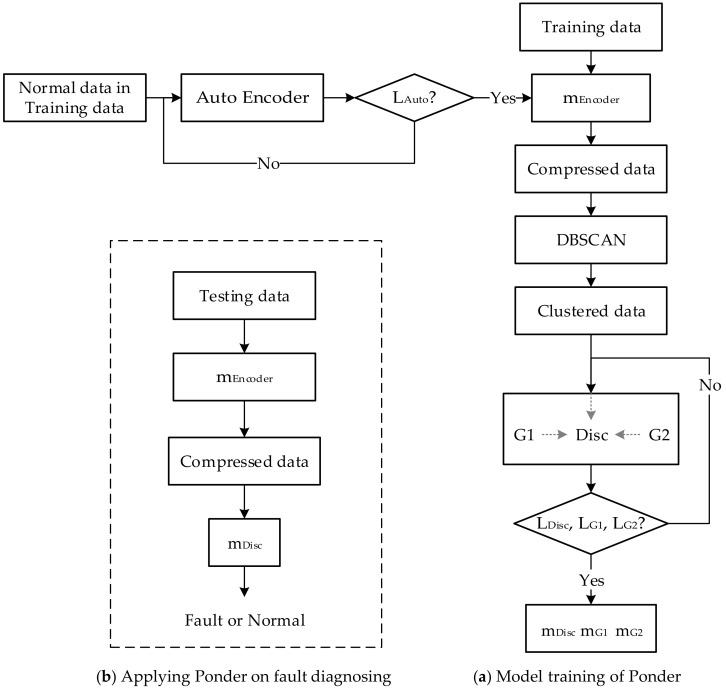
The work-flow of Ponder’s building and diagnosing.

*Implementation of*Ponder. Following each subsection including hyper-parameters of Section 4.2, the network details (e.g., neural network’s layer number and types) can be obtained, and the network model (with network parameters) could be trained by minimizing four losses (i.e., $L_{auto}$, $L_{G1}$, $L_{G2}$, and $L_{dis}$) with the Adam optimizer. We show the work-flow of building Ponder and that of applying Ponder to fault diagnosing in Figure 3.

*Detailed Evaluation.* Based on the above setup, we aim to further refine the evaluation aspects into the following four questions:

$Q1_{a}$: How is the performance of Ponder compared to that of DMO under the circumstance of little data?

$Q2_{b}$: How is the performance of Ponder compared to existing diagnosing models?

$Q3_{c}$: How does Ponder behave over time?

$Q4_{d}$: How much overhead does the Ponder introduce to the diagnosing model?

### 5.2. Experimental Design and Results

In this section, we aim to answer each question in detail. For each question, we will first describe the experiment design if necessary, then the results.

$Q1_{a}$: How is the performance of Ponder compared to that of DMO under the circumstance of little data?

To validate the design choice of two generators, we compare the performance of Ponder with that of only one generator. Their results are shown in Table 1. At a high level, we can see that Ponder outperforms that of one generator on two datasets. Concretely, the F1-score of Ponder on the test dataset arrives at 0.950, while models that generate fault data with only one generator have the F1-score of 0.931 and 0.927. The performance on D-Feb. has the same conclusion and is omitted for brevity. These results all indicate that Ponder’s two generators are better than that with one generator for fault data generating.

$Q2_{b}$: How is the performance of Ponder compared to existing diagnosing models?

We compare the performance of Ponder, RF, and ODDS on dataset D-Feb to evaluate the performance of Ponder. Their results are shown in Table 2. We can see that Ponder outperforms all the baseline models. More specially, Ponder has an F1-score of 0.93, while RF has an F1-score of 0.83. The reason for RF’s smaller results might be the lack of future distribution of fault data. ODDS detects fault samples with the F1-score of 0.91. The explanation for the lower F1-score of ODDS might be the assumption that the fault data in outlier are scattered randomly.

In addition, we also notice that Ponder diagnoses fault with the lowest false positive rate. The lowest FPR means that there are less false diagnoses where normal state is regarded as false state. These results all illustrate that Ponder takes the characteristic of fault data and helps to improve the performance of existing fault diagnosing methods.

$Q3_{c}$: How does Ponder behave over time?

To evaluate the Ponder’s robustness over time, we compare the performance of Ponder on D-Feb and D-Oct. In addition, we also give the results of RF and ODDS as a comparison. Their results are shown in Table 2. We can see that Ponder does not have obvious degradation on D-Oct. However, RF and ODDS detect fault data with little decline. More specifically, the F1-score of Ponder declines by 2%, and the baseline models of RF and ODDS decline by 4%.

As we do not have more data to validate the degradation over time, we verify that, on these three datasets, Ponder behaves better over time. This demonstrates that Ponder is robust as time goes by.

$Q4_{d}$: How much overhead does the Ponder introduce to the diagnosing model?

We record the average fault detection time that Ponder spends on data processing and fault detection for each sample in D-Oct. We do not consider the time spent on training each model, since these models are trained offline and the training time could be compensated by a large amount of data. As a comparison, we also give the results of a baseline model. The detecting time of each model is shown in the last column of Table 2. We can see that the average detection time of Ponder models is comparable to that of baseline models; in particular, it is more similar to that of the ODDS model.

## 6. Discussion

*Static vs. Learning based Model.* Static diagnosing has high precision but with expensive manual cost. In cases without strong coupling, static techniques such as domain-expertise or a rule based method is a better choice. For cases where strong coupling exists among components, learning based methods have superiority. Ponder is a kind of learning based diagnosing method for biased data.

*Real-time Diagnosing.* Even though Ponder is evaluated on the offline data, it can be used for real-time diagnosing. It is possible to transform software codes into hardware language and implemented it with hardware. We will leave the further hardware implementation to future work.

*Limitations.*Ponder works under an assumption that data in normal data are stable. However, actually normal data may change with time going, for example, a substituted component that has the same function with original but with different data expressing. Thus, Ponder needs to be updated for a certain of time. In addition, to determine when to update the diagnosing model, authors in [37] have introduced methods.

## 7. Related Works

*Anomaly Diagnosis.* Anomaly diagnosis techniques diagnose fault state with only normal data. They make an assumption that normal data are stable and unchanged for a period. In our work, we make the same assumption, while the difference is that we take full advantage of the little fault data. We do not use it as our baseline since it has been verified by these works [33].

*Data Augmentation.* Data augmentation is a technique derived from computer domains and usually used for complementing the needed data. It usually achieves its goal via a generative adversarial network. Current data augmentation works mainly use it to generate data based on existing data distribution or learn an unknown distribution with existing data distribution. The most related work to ours is [33]. In Ref. [33], the authors design two generators for assisting bot data generation based on two different assumptions. However, we still use two generators but with a different manner. We assume that the fault data that need to be generated conform to the same assumption (i.e., dense at fault clusters or outliers and sparse at normal clusters or outliers). In addition, the application domain is essentially different. We do not consider other related works in terms of auto-encoder and clustering, as the two techniques are just an assist for our data generation and have no difference from others.

## 8. Conclusions

This paper presents Ponder, a fault diagnosing model for BS-UAV’s taking off with little data. By designing novel generators for learning fault data’s distribution, we generate plenty of fault data and enable a deep learning based fault detection model to diagnose BS-UAV’s take off timely.

## Figures and Tables

**Table 1:** F1-score comparison of Ponder models with different numbers of generators.

Dataset	G1 Generator	G2 Generator	Both Generators
Testing	0.931	0.927	0.950
D-Feb.	0.917	0.911	0.932

**Table 2:** Performance comparison of Ponder models and baseline models on two datasets.

Dataset	D-Feb				D-Oct				
Metric	Precision	Recall	F1-Score	FPR	Prec.	Rec.	F1.	FPR	Time (ms)
RF	0.82	0.84	0.83	0.08	0.78	0.79	0.79	0.08	0.17
ODDS	0.90	0.92	0.91	0.06	0.88	0.87	0.87	0.06	0.11
Ponder	0.92	0.94	**0.93**	0.04	0.90	0.91	**0.91**	0.04	0.12

## Data Availability

Not applicable.
